# Premenopausal Patients With Clinically Aggressive Metastatic Breast Cancer Successfully Treated With a First‐Line Palbociclib‐Containing Regimen: Two Cases and Literature Review

**DOI:** 10.1002/cnr2.70417

**Published:** 2025-12-03

**Authors:** Pei‐An Fu, Ya‐Chun Hsu, Hui‐Ping Hsu, Tzu‐Chien Lin, Wei‐Pang Chung

**Affiliations:** ^1^ Division of Hematology, Department of Internal Medicine National Cheng Kung University Hospital, College of Medicine, National Cheng Kung University Tainan Taiwan; ^2^ Department of Radiology National Cheng Kung University Hospital, College of Medicine, National Cheng Kung University Tainan Taiwan; ^3^ Department of Surgery National Cheng Kung University Hospital, College of Medicine, National Cheng Kung University Tainan Taiwan; ^4^ Division of Hematology and Oncology, Department of Internal Medicine Kaohsiung Veterans General Hospital Kaohsiung Taiwan; ^5^ Department of Oncology National Cheng Kung University Hospital, College of Medicine, National Cheng Kung University Tainan Taiwan; ^6^ Center for Applied Nanomedicine National Cheng Kung University Tainan Taiwan

**Keywords:** CDK4/6 inhibitor, endocrine therapy, hormone receptor‐positive, metastatic breast cancer, palbociclib

## Abstract

**Background:**

Palbociclib, which is an oral small‐molecule cyclin‐dependent kinase 4 and 6 (CDK4/6) inhibitor, demonstrated its efficacy in hormone receptor‐positive (HR+) and human epidermal growth factor receptor 2‐negative (HER2−) metastatic breast cancer together with endocrine therapy. However, patients with visceral crises (symptomatic visceral dissemination) are recommended to consider chemotherapy rather than endocrine‐based therapy. Currently, more reports and evidence support that patients with aggressive visceral metastasis can be effectively managed using both a CDK4/6 inhibitor and endocrine therapy.

**Case:**

We report two premenopausal patients with HR+/HER2− metastatic breast cancer, whose diseases diffusely involved major organs and were successfully managed with palbociclib and endocrine therapies as the initial therapy.

**Conclusion:**

In the two cases we reported, first‐line palbociclib therapy shows adequate and timely responses for premenopausal HR+/HER2− metastatic breast cancer patients. Although not widely utilized, frontline therapy with palbociclib combined with endocrine treatments may be a choice for HR+/HER2− metastatic breast cancer patients experiencing severe visceral metastasis.

## 1 Introduction

Palbociclib is an oral cyclin‐dependent kinase 4 and 6 (CDK4/6) inhibitor that has been documented to slow breast cancer cell growth with estrogen receptors and can also work well with anti‐estrogen drugs [1]. The pivotal PALOMA‐2 study assigned first‐line treatment to menopausal females with hormone receptor‐positive (HR+) and human epidermal growth factor receptor 2‐negative (HER2−) metastatic breast cancer. Those patients receiving letrozole plus palbociclib had significantly longer progression‐free survival (PFS) than patients receiving letrozole alone (24.8 vs. 14.5 months; hazard ratio [HR]: 0.58) [2]. The phase 3 MONALEESA‐7 trial confirmed the survival advantages of premenopausal patients using goserelin, either tamoxifen or a nonsteroidal aromatase inhibitor, and ribociclib [3, 4]. These large‐scale studies revealed that both premenopausal and postmenopausal patients benefited from the combined CDK4/6 inhibitors and endocrine therapies. However, patients with symptomatic visceral metastases at risk for life‐threatening complications were excluded from the PALOMA‐2 trial, and those with inadequate organ functions were excluded from the MONALESSA‐7 trial. The efficacy of endocrine‐based regimens with CDK4/6 inhibitors against these aggressive diseases was unclear until the findings of the RIGHT Choice study [5]. In the study, goserelin, either letrozole or anastrozole, and ribociclib were better than combination chemotherapy in terms of PFS for premenopausal patients having aggressive HR+/HER2− advanced breast cancer. Hence, we present two premenopausal patients with HR+/HER2− metastatic breast cancer, whose advanced visceral metastases were successfully managed by endocrine therapies plus palbociclib.

## 2 Case 1

**FIGURE 1:**
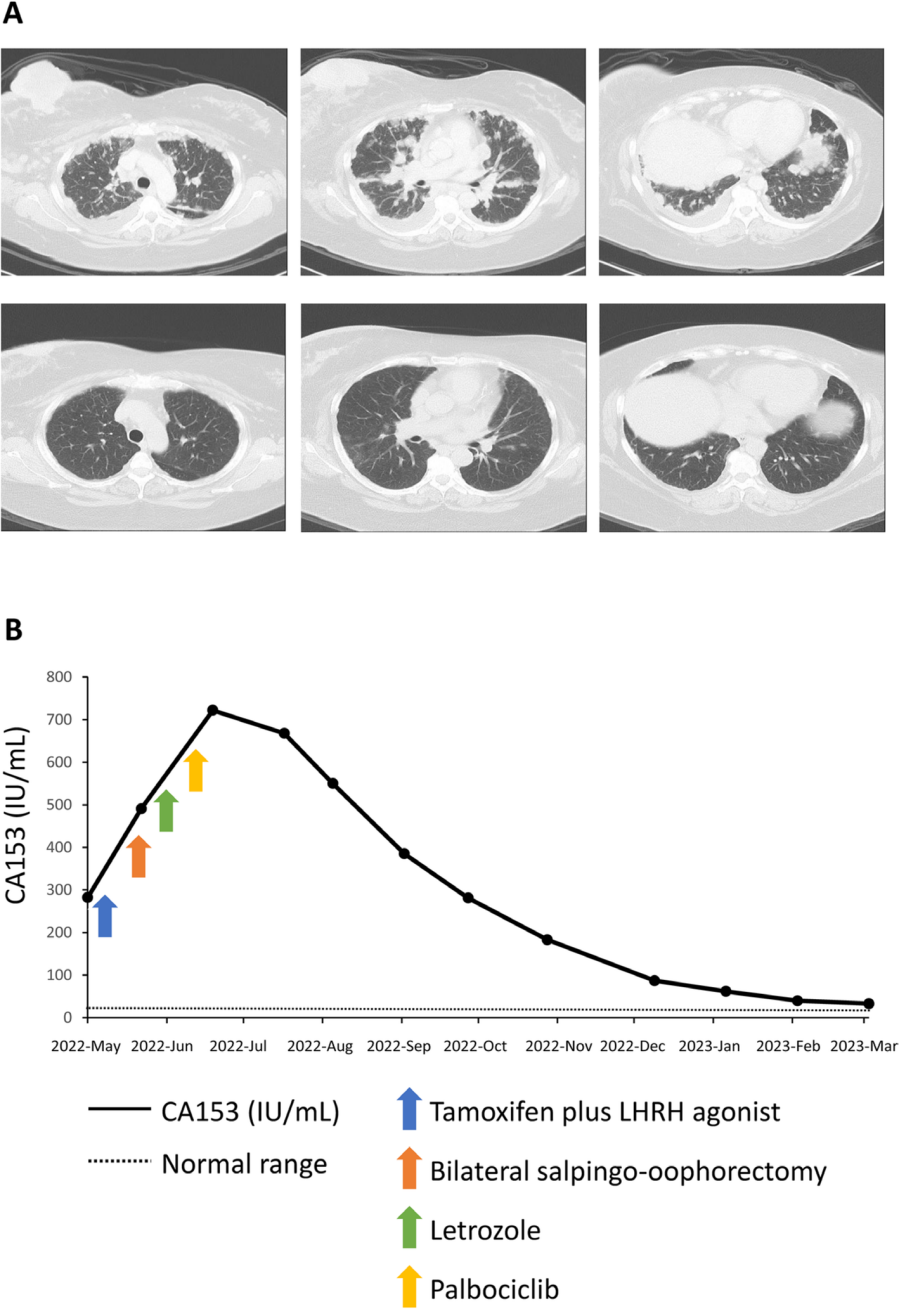
Changes in lung metastases and CA153 levels after palbociclib and letrozole treatment in Case 1. (A) Chest computed tomography of Case 1 showed right breast cancer with bilateral lung metastases and pleural seeding at diagnosis. The top panels depict the disease status before palbociclib and letrozole treatment, while the bottom panels show marked tumor regression 6 months after treatment. (B) Trends in CA153 levels in Case 1 and the timing of interventions.

A 48‐year‐old premenopausal female patient presented with a huge right breast mass accompanied by a chronic productive cough, and visited National Cheng Kung University Hospital in Taiwan. Pathology confirmed invasive ductal carcinoma after a right breast tumor incisional biopsy. Immunohistochemical (IHC) stain revealed estrogen receptor (ER)‐positive (> 95% tumor cells), progesterone receptor (PR)‐positive (> 95% tumor cells), and HER2‐negative with an IHC stain of 0. Ki‐67 score was 25%–30%. Chest computed tomography (CT) revealed diffuse lung metastases and pleural seedings (Figure 1A). We immediately initiated tamoxifen (20 mg per day) and leuprorelin (3.75 mg every month) treatment after the diagnosis. A few days later, a bilateral salpingo‐oophorectomy was performed, as the patient's condition was stable. Subsequently, the endocrine therapy was changed to letrozole (2.5 mg per day). Palbociclib (125 mg once a day) was added once available. The palbociclib was prescribed for 21 days in each 28‐day cycle. Only mild leukopenia was noted, without any opportunistic infection. Her cough resolved with the endocrine‐based treatment after 6 months. A follow‐up CT revealed significant improvement in lung metastases (Figure 1A), and CA153 tumor marker levels decreased from a peak of 722 U/mL to a low of 32.9 U/mL (normal range of CA153: ≤ 26.2 U/mL) (Figure 1B).

## 3 Case 2

**FIGURE 2:**
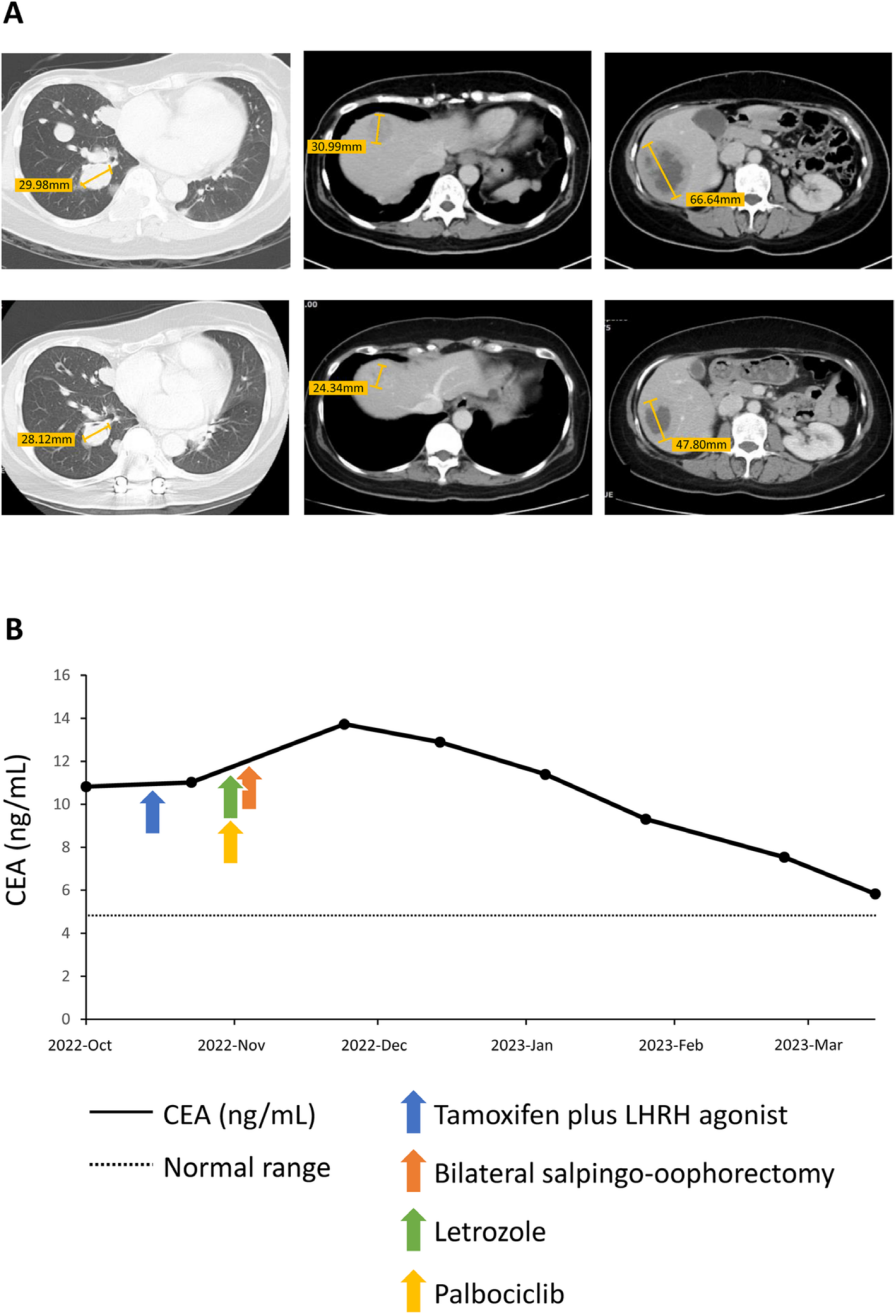
Changes in liver metastasis and CEA level after palbociclib and letrozole treatment in case 2. (A) Chest and abdominal computed tomography of case 2 demonstrated multiple pulmonary and hepatic metastases at recurrence. The top panels show the disease status prior to palbociclib and letrozole treatment, whereas the bottom panels illustrate marked regression of liver metastases 6 months after therapy. (B) Serial changes in carcinoembryonic antigen (CEA) levels in Case 2 in relation to the treatment timeline.

A 43‐year‐old premenopausal female patient presented with a right breast mass for 1 month. She underwent a total mastectomy and sentinel lymph node dissection in National Cheng Kung University Hospital. The pathology confirmed invasive ductal carcinoma, which revealed ER‐positive (95% tumor cells), PR‐negative, and HER2‐negative with an IHC stain of 0. The Ki‐67 score was 14%. She received 6 cycles of adjuvant chemotherapy with fluorouracil (500 mg/m^2^), epirubicin (75 mg/m^2^), and cyclophosphamide (500 mg/m^2^), subsequently using tamoxifen. Diffuse lung and liver metastases were shown by the CT 2 years postoperatively (Figure 2A), along with bone metastasis. Further, the pathology of the excised paraspinal soft tissue confirmed ER‐positive (95% tumor cells), PR‐negative, and HER2‐negative metastatic carcinoma. Tamoxifen (20 mg per day) and leuprorelin (3.75 mg every month) treatment had been initiated, followed by letrozole (2.5 mg once a day) plus palbociclib (125 mg once a day) after she underwent a bilateral salpingo‐oophorectomy. Grade 4 neutropenia was noted, so palbociclib was tapered down to 100 mg for 2 weeks, followed by 2 weeks of rest. Carcinoembryonic antigen (CEA) levels dropped from 10.82 to 6.26 ng/mL (normal range of CEA level: 0.0–4.6 ng/mL) (Figure 2B). A follow‐up CT in 6 months confirmed interval regression of lung and liver metastases (Figures 2A).

## 4 Discussion

Visceral crisis describes patients with excessive tumor burden that causes severe organ dysfunction, such as diffuse liver metastasis, diffuse pulmonary metastasis, meningeal spreading, and so forth. Patients with metastatic breast cancer having visceral crises demonstrated a shorter survival and a dismal outcome and pose a tremendous challenge for physicians. A CDK4/6 inhibitor, such as palbociclib, demonstrated its efficacy in patients with treatment‐naive HR+/HER2− breast cancer experiencing visceral metastases [2], but those having visceral crises or aggressive visceral metastasis were excluded from the trial. The guidelines recommend chemotherapy rather than a CDK4/6 inhibitor along with endocrine therapy in patients having visceral crises due to a lack of large‐scale studies supporting [6, 7]. The response time is one of the concerns for using CDK4/6 inhibitor‐based regimens in such a population. The response to endocrine therapy is believed to be slower than that to chemotherapy. However, this notion has been contested in the era of CDK4/6 inhibitors. Almost half of the patients participating in the PALOMA‐2 trial, who were administered palbociclib alongside letrozole, exhibited an objective response within 3 months [8]. The post hoc analysis revealed that CDK4/6 inhibitors demonstrated rapid efficacy for patients with metastatic breast cancer, even in cases involving visceral disease.

Not only has the response time been questioned for the adoption of CDK4/6 inhibitors in patients experiencing visceral crises, but also the PFS when compared to chemotherapy. A phase 2 Young‐PEARL trial evaluating the efficacy of palbociclib plus endocrine therapy and capecitabine in premenopausal patients with HR+/HER2− metastatic breast cancer whose diseases were resistant to tamoxifen revealed that palbociclib demonstrated superior PFS (20.1 vs. 14.4 months) [9]. However, the phase 3 PEARL trial analyzing the effectiveness of palbociclib plus endocrine therapy and capecitabine in postmenopausal patients with aromatase inhibitor‐resistant HR+/HER2− metastatic breast cancer showed that endocrine‐based therapy was not more beneficial than capecitabine [10]. The contradictory findings of these two studies indicate that menopausal status, ethnicity, or previous endocrine therapy may influence the comparative efficacy of palbociclib along with endocrine therapy and chemotherapy. Accordingly, frontline palbociclib with endocrine therapy is still considered for HR‐positive metastatic breast cancer, especially for patients unfit for chemotherapy.

Several studies are currently examining the efficacy of first‐line CDK4/6 inhibitors for metastatic breast cancer patients having aggressive visceral metastasis, in light of the improved outcomes brought by these inhibitors. The phase 2 RIGHT Choice study enrolled patients having treatment‐naive premenopausal HR+/HER2− aggressive metastatic breast cancer, with > 50% of patients having visceral crises [5]. The ribociclib plus endocrine treatment had a numerically longer response time compared to physicians' choice of combination chemotherapy (4.9 vs. 3.2 months; HR: 0.78; 95% confidence interval: 0.56–1.09), although it may be expected. Eventually, the study demonstrated that ribociclib plus endocrine therapy surpassed combination chemotherapy in terms of PFS (24.0 vs. 12.3 months; HR: 0.54; *p*‐value: 0.0007), indicating that these patients should be treated with CDK4/6 inhibitor‐based regimens. As far as we know, there have been no reported clinical trials of using frontline palbociclib with endocrine treatment for patients experiencing aggressive visceral metastasis.

Palbociclib is a CDK4/6 inhibitor associated with lower rates of diarrhea and hepatic toxicity compared to other agents in its class, resulting in better tolerability and improved patient compliance. Here, we presented two premenopausal female patients with substantial tumor burdens and visceral crises. Sequential administration of endocrine therapies, bilateral salpingo‐oophorectomy, and palbociclib after close monitoring for aggressive visceral metastasis stabilized their disease. Our experiences support the effective treatment of patients with aggressive visceral crises with a combined usage of palbociclib and endocrine therapy.

## Author Contributions

Pei‐An Fu contributed to the manuscript draft. Wei‐Pang Chung revised the manuscript critically for important intellectual content. Ya‐Chun Hsu, Hui‐Ping Hsu, Tzu‐Chien Lin, and Wei‐Pang Chung contributed to the data collection and patient care. Pei‐An Fu, Wei‐Pang Chung, Ya‐Chun Hsu, Hui‐Ping Hsu, and Tzu‐Chien Lin all read and approved the final manuscript.

## Funding

The authors have nothing to report.

## Ethics Statement

All subjects gave their informed consent for inclusion. The study was conducted in accordance with the Declaration of Helsinki, and the protocol was approved by the Ethics Committee of the Institutional Review Board, National Cheng Kung University (B‐EC‐112‐006).

## Consent

Written informed consent was obtained from the patients for the publication of case details and use of images.

## Conflicts of Interest

The authors declare no conflicts of interest.

## Data Availability

The data that support the findings of this study are available on request from the corresponding author. The data are not publicly available due to privacy or ethical restrictions.
